# Chemotherapy aNd chemoradiotherapy for adenocarcinoma of the OESophagus and esophagogastric junction with oligometastases: Protocol of the TNT-OES-1 trial

**DOI:** 10.1016/j.conctc.2022.100934

**Published:** 2022-05-28

**Authors:** Charlène J. van der Zijden, Ben M. Eyck, Ate van der Gaast, Leni van Doorn, Joost J.M.E. Nuyttens, J. Jan B. van Lanschot, Bas P.L. Wijnhoven, Bianca Mostert, Sjoerd M. Lagarde

**Affiliations:** a Department of Surgery, Erasmus MC Cancer Institute, Erasmus University Medical Center, Rotterdam, the Netherlands; b Department of Medical Oncology, Erasmus MC Cancer Institute, Erasmus University Medical Center, Rotterdam, the Netherlands; c Department of Radiation Oncology, Erasmus MC Cancer Institute, Erasmus University Medical Center, Rotterdam, the Netherlands

**Keywords:** Esophageal cancer, Esophagogastric junction, Adenocarcinoma, Oligometastatic disease, Chemotherapy, Chemoradiotherapy

## Abstract

**Background:**

FLOT and CROSS are effective neoadjuvant regimens for esophageal cancer patients. Chemotherapy (FLOT) is aimed to have merely a systemic effect whereas neoadjuvant chemoradiotherapy (CROSS) achieves good locoregional response with clinically complete response (cCR) rates up to 33% [1]. The aim of the present study is to assess safety and feasibility of dual therapy (FLOT-CROSS) in patients with oligometastases.

**Methods:**

This phase-II single-center, single-arm, intervention study includes patients with oligometastatic adenocarcinoma of the esophagus or esophagogastric junction. Patients will be treated with four biweekly cycles of FLOT, consisting of intravenous fluorouracil (2600 mg/m^2^), leucovorin (200 mg/m^2^), oxaliplatin (85 mg/m^2^) and docetaxel (50 mg/m^2^). Response evaluation by CT-scan will be performed 4–6 weeks after completion of FLOT. In case of regression or stable disease according to RECIST criteria (v.1.1), patients will receive additional CROSS, consisting of five weekly cycles of intravenous carboplatin (AUC 2) and paclitaxel (50 mg/m^2^), with concurrent 41.4 Gy radiotherapy, in 23 daily fractions of 1.8 Gy [2]. Response evaluation by endoscopy with biopsies, endoscopic ultrasonography and CT-scan will be performed 4–6 weeks after completion of CROSS. Primary endpoint is tolerability of FLOT-CROSS, defined as the proportion of patients who complete the full regimen. Secondary endpoints include disease control rate, objective response rate, overall survival and progression-free survival. In total, 20 patients will be included.

**Discussion:**

If patients are able to complete and tolerate FLOT-CROSS, this regimen should be tested in a phase-III trial and as neoadjuvant treatment in patients with locally advanced non-metastatic esophageal or junctional adenocarcinoma.

## 1 Background

Adenocarcinoma of the esophagus or esophagogastric junction (EGJ) is an aggressive cancer with 85.000 new cases worldwide every year [1]. In case of locally advanced non-metastatic tumors, patients can be treated with curative intent. Approximately half of patients present with distant metastases and only palliative treatment can be offered [2]. Oligometastastic disease, defined as abdominal or retroperitoneal lymph node metastases in one curable organ site only without clinically visible or symptomatic carcinomatosis of the peritoneum or pleura, can be treated by peri-operative chemotherapy with fluorouracil, leucovorin, oxaliplatin and docetaxel (FLOT) followed by optional surgical resection [3].

In the Netherlands and across most countries in Europe, standard potentially curative treatment for patients with non-metastatic advanced adenocarcinoma of the esophagus or EGJ consists of peri-operative chemotherapy or neoadjuvant chemoradiotherapy (nCRT). This chemoradiotherapy regimen combines carboplatin and paclitaxel with concurrent 41.4 Gy radiotherapy (CROSS regimen), followed by esophagectomy [4]. While CROSS prior to surgery improved 10-year survival from 25% to 38%, CROSS mainly affects locoregional control [5]. This is reflected by the microscopically radical (R0) resection rate of 92%, pathologically complete response rate (pCR) of 23% and decreased risk of isolated locoregional relapse of 8% at 10 year follow up (HR 0.39, 95% CI 0.21–0.72) in favor of chemoradiotherapy over surgery alone [4,6]. CROSS is well-tolerated and can be given in an outpatient setting. Carboplatin and paclitaxel are mainly radiosensitizers with limited systemic effects although it also seems to have a beneficial effect to distant progression [7].

Since the FLOT-4 trial has shown that FLOT, compared to the previous standard therapeutic regimen of cisplatin, epirubicin and capecitabine (MAGIC) resulted in a substantially improved survival (HR 0.77, 95% CI 0.63–0.94), the standard of care for patients with adenocarcinoma of either the EGJ (invading the cardia >4 cm) or stomach in most European countries changed to peri-operative chemotherapy with FLOT, combined with surgical resection [8]. The subgroup of patients with junctional adenocarcinoma treated with FLOT had respective 2-year and 5-year overall survival of 65% and 39%, which seems comparable to the CROSS trial. Peri-operative FLOT chemotherapy has a significant effect on reducing the risk of distant metastases resulting in a median disease-free survival (DFS) of 30 months [8]. The locoregional effect of FLOT, however, seems less pronounced than that of the CROSS regimen, which is reflected by the lower R0 resection rate and lower pCR rate [9,10]. The interim analysis of the Neo-AEGIS trial comparing the FLOT and CROSS regimens shows that peri-operative chemotherapy is non-inferior to chemoradiotherapy in patients with adenocarcinoma of the esophagus or EGJ [11].

The combination of potent systemic chemotherapy with a well-tolerated and locoregionally effective chemoradiotherapy regimen may be an attractive treatment option in patients with oligometastatic adenocarcinoma of the esophagus or EGJ. Several other studies combining chemotherapy with chemoradiotherapy show an improved response compared to chemotherapy or chemoradiotherapy alone [[12], [13], [14]]. These regimens, however, either use outdated regimens with low response rates and substantial toxicity.

Therefore, the aim of the present phase II study is to assess the safety, feasibility and clinical response of FLOT-CROSS.

## 2 Methods

### 2.1 Study design and recruitment

The TNT-OES-1 trial is a phase-II, prospective, single-center, single-arm intervention study. The study will be conducted at the Erasmus MC Cancer Institute. The study is approved by the local Ethics Committee (Erasmus University Medical Center Rotterdam; MEC 2020-0747) and registered in the Dutch Trial Registry (number NL9269) [15]. All patients will provide written informed consent prior to start of the study. The study will be performed in accordance with the Declaration of Helsinki (64th World Medical Association General Assembly, Fortaleza, Brazil, October 2013).

### 2.2 Eligibility criteria

Patients between 18 and 75 years who have been diagnosed with adenocarcinoma of the esophagus or EGJ (type 1 and type 2 according to Siewert classification of esophagogastric adenocarcinoma) with a resectable primary tumor in combination with oligometastatic disease will be recruited for this study [16]. Patients will be considered eligible according to the following criteria.

Inclusion criteria:

1. Histologically-proven cT1N1-3M1 or cT2-4aN0-3M1 adenocarcinoma of the esophagus or EGJ according to the 8th edition of the Union for International Cancer Control (UICC) TNM classification for Esophageal Cancer [17];
2. Oligometastatic disease, which is defined as a maximum of four resectable metastatic lesions or lesions suitable for stereotactic irradiation. These four lesions can be present in a maximum of two organs (liver, lung, bones or adrenal gland). Lymph nodes are not counted as an organ. If metastatic retroperitoneal or supraclavicular lymph nodes are present, this lymph node site counts as one metastatic lesion, and together with the possible metastases in organs cannot exceed the number of four lesions.
3. No prior abdominal, thoracic or cervical radiotherapy overlapping with the CROSS irradiation fields;
4. No prior cytotoxic chemotherapy
5. Eastern Cooperative Oncology Group (ECOG) Performance Status of 0 or 1 [18];
6. Adequate cardiac and respiratory function assessed by standard electrocardiogram and additional diagnostics in case of cardiopulmonary complaints or comorbidity (*i.e.* lung function test or echocardiography);
7. Adequate bone marrow function (white blood cells >3x10^9/L; hemoglobin >5.5 mmol/L; platelets >100x10^9/L). In the event of transfusions, the last red blood cell transfusion should be more than two weeks before inclusion;
8. Adequate renal function (glomerular filtration rate >50 mL/min or serum creatinine ≤1.5x upper level of normal (ULN)) and adequate liver function (total bilirubin <1.5x ULN, aspartate transaminase (AST) < 2.5x ULN and alanine transaminase (ALT) < 3x ULN);
9. A negative serum pregnancy test in women of child-bearing potential during screening period;
10. Use of adequate contraception during the study and for three months after the end of the study;

Exclusion criteria are:

1. Patients with overt peritoneal or pleural dissemination, as detected on PET-CT or regular CT-scan. In patients in whom a diagnostic laparoscopy is indicated (to assess gastric involvement and the possibility to perform gastric tube reconstruction or to exclude peritoneal disease), tumor-positive cytology peritoneal fluid is also an exclusion criteria;
2. Gastric carcinoma or Siewert 3 (bulk of tumor below EGJ) [16];
3. Clinically significant (active) cardiac disease (e.g. symptomatic coronary artery disease of myocardial infarction within the last 12 months) or lung disease (forced expiratory volume in 1 s (FEV1) <1.5L)
4. Peripheral neuropathy grade more than 1, according to the Common Terminology Criteria for Adverse Events (CTCAE) version 5.0 [19];
5. Pregnant and lactating women, or patients of reproductive potential who are not using effective contraception. If barrier contraceptives are used, they must be continued by both sexes throughout the study;
8. Secondary primary cancer with the exclusion of basal cell carcinoma of the skin;

## 3 Study process

**Fig. 1:**
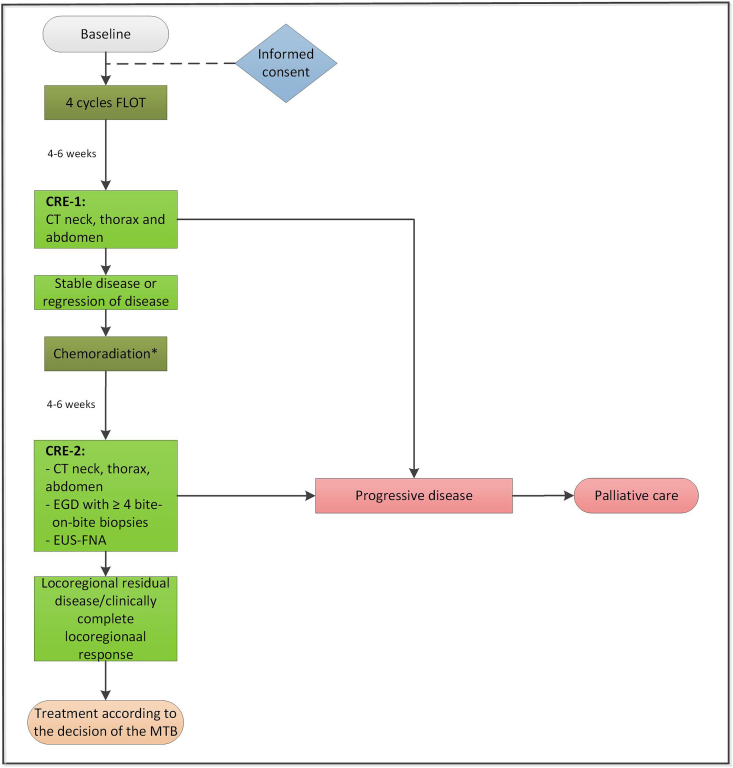
Flowchart of the TNT-OES-1 trial FLOT: combination of fluorouracil, leucovorin, oxaliplatin and docetaxel; CRE: clinical response evaluation; PET-CT: positron emission tomography; EGD: esophagogastroduodenoscopy; EUS: endoscopic ultrasound; FNA: fine-needle aspiration; MTB: multidisciplinary tumor board. *Chemoradiation consists of five weekly cycles carboplatin/paclitaxel with concurrent radiotherapy (CROSS).

An overview of the study flow is presented in Fig. 1.

### 3.1 Baseline examination and inclusion

Prior to study inclusion, patients undergo standard of care work-up including esophagogastroduodenoscopy (EGD) with conventional tumor biopsies, endoscopic ultrasonography (EUS) in case of a traversable tumor, high resolution CT-scan of the thorax, abdomen and pelvis and whole-body PET-CT to stage the tumor and look for possible dissemination. If indicated, an additional MRI of the liver and/or a diagnostic laparoscopy will be performed. Furthermore, quality of life (QoL) questionnaires will be obtained at baseline. Written informed consent for study participation will be obtained from eligible patients after baseline diagnosis and staging. An overview of the study assessments is presented in Table 1.

**Table 1:** Schedule of assessments.

Required Investigations	Pre-treatment	FLOT chemotherapy (each cycle)	CRE-1	Chemoradiotherapy (each chemo cycle)	CRE-2
Eligibility check	x^a^				
Written informed consent	x^a^				
Inclusion	x				
Medical history incl. AEs	x^a^	x	x	x	x
Physical exam^b^	x^a^	x	x	x	x
Weight and vital signs	x	x	x	x	x
ECOG performance status	x^a^	x	x	x	x
QoL QLQ-C30 and QLC-OG25	x		x		x
Haematology^c^	x^a^	x	x	x	x
Biochemistry^d^	x^a^	x	x	x	x
PET-CT	x^a^				
CT thorax, abdomen and pelvis (4 phase)	x^a^		x		x
EGD + EUS^e^	x^a^				x
Pulmonary function tests^f^	x^a^				
ECG	x^a^				
Placement of PICC	x^a^				
Toxicity^g^	x^a^	x	x	x	x
Surgery	On indication^h^				

FLOT: combination of fluorouracil, leucovorin, oxaliplatin and docetaxel; CROSS: combination of carboplatin/paclitaxel and radiotherapy; CRE: clinical response evaluation; AEs: adverse events; ECOG: Eastern Cooperative Oncology Group; QoL: quality of life; QLQ: quality of life questionnaire; PET: positron emission tomography; EGD: esophagogastroduodenoscopy; EUS: endoscopic ultrasound; ECG: electrocardiogram; PICC: peripherally inserted central venous catheter.

a Standard palliative treatment assessments.

b Full physical examination at pre-treatment, on indication on subsequent visits.

c Haemoglobin, Leukocytes, Neutrophils, Thrombocytes, MCV, RDW.

d Alkaline phosphatase, AST, ALT, gammaGT.

e Radial EUS with measurement of maximum tumor thickness and –area. Linear EUS: with FNA of any suspected lymph and minimum of 8 biopsies including 4 bite-on-bite biopsies.

f Only on indication.

g To be evaluated after each cycle, according to CTCAE v5.0.

h Diagnostic laparoscopy on indication, if indicated combined with placement of feeding jejunostomy.

### 3.2 Systemic treatment

In case of significant dysphagia prior to systemic therapy, patients will be treated according to the local protocol (*i.e.* placement of a stent or duodenal tube). All patients will start with four cycles of FLOT chemotherapy according to the standard schedule [8]. This regimen consists of 2-weekly cycles of 5-fluorouracil intravenous (iv) 2600 mg/m^2^ in a 24 h infusion, docetaxel iv at a dose of 50 mg/m^2^, oxaliplatin iv at a dose of 85 mg/m^2^ and leucovorin iv at a dose of 200 mg/m^2^. Expected adverse effects of FLOT chemotherapy mainly consist of neutropenic fever, mucositis and peripheral neuropathy. These effects will be managed according to local protocols. If an adverse effect is present or the blood test shows neutropenia, a dose reduction may be considered or the next cycle of chemotherapy may be postponed for one or two weeks. In case of postponed treatment, this will be registered as a protocol deviation but not as study drop-out. In case of dose-limiting toxicities (DLTs, judgement of the treating physician) a dose reduction to 75% of the initial dose of docetaxel and/or oxaliplatin is warranted. Subsequent DLTs lead to a further dose reduction to 50% of the initial dose level. If after dose reduction to 50% DLTs re-occur, treatment within the trial is stopped for that patient. In the event of treatment delay due to toxicities for more than three weeks, patients also go off-study.

### 3.3 CRE-1

A first clinical response evaluation (CRE-1) will take place 4–6 weeks after completion of the last cycle of FLOT chemotherapy, consisting of a CT-scan of the neck, thorax and abdomen (Table 1). Only in case of stable disease or regression of the disease according to RECIST criteria (version 1.1), patients will be eligible for continuation in the study [20]. Patients with progressive disease will go off-study and will be treated according to the decision of the multidisciplinary tumor board (e.g. second line systemic treatment and/or best supportive care).

### 3.4 Chemoradiotherapy

Patients without progressive disease will continue within the study and will be treated with chemoradiotherapy according to the CROSS regimen [21]. This regimen consists of five weekly cycles of carboplatin iv at an area under the curve (AUC) of 2 mg/ml/min and paclitaxel iv at dose 50 mg/m^2^ at the first day of each week, with concurrent radiotherapy 41.4 Gy given in 23 fractions of 1.8 Gy for five days per week, starting at the first day of each cycle of chemotherapy.

### 3.5 CRE-2

The second clinical response evaluation (CRE-2) will be performed 4–6 weeks after completion of the last session of chemoradiotherapy, using a combination of EGD with at least 4 bite-on-bite biopsies, EUS with fine-needle aspiration (FNA) of suspected lymph nodes and CT-scan of the neck, thorax and abdomen. When taking bite-one-bite biopsies, a second biopsy is taken exactly at the same location of the first biopsy which increase the change of detecting submucosal tumor deposits. If suspicious lesions are present, bite-on-bite biopsies will be taken of these lesions, always in combination with random bite-on-bite biopsies in the area of the primary tumor.

### 3.6 Follow-up

Further treatment and follow-up will be determined in a multidisciplinary tumor board and will not be considered part of the investigational treatment. Briefly, several further treatment strategies can be considered. Patients with progressive disease may be treated with second line systemic therapy and/or best supportive care. Patients who have stable disease or regression of disease according to the RECIST criteria (version 1.1), will be eligible for continuation of another maximum of four FLOT chemotherapy courses after completion of chemoradiotherapy according to the CROSS regimen [20]. The primary tumor and/or distant metastases may be treated using local therapy (radiofrequency ablation, microwave ablation or surgical metastasectomy) or patients may undergo active surveillance when no residual tumor is detected. If locoregional regrowth without further distant dissemination is detected during active surveillance, esophagectomy might be offered in selected cases.

### 3.7 Quality of life assessments

QoL of patients enrolled in this trial will be evaluated since an intensive treatment consisting of chemotherapy followed by chemoradiotherapy and possible surgery is expected to influence QoL. To evaluate the well-being of patients, two of the European Organization for Research and Treatment of Cancer (EORTC) QoL questionnaires will be used: the EORTC QLQ-C30 for general cancer-related QoL and the EORTC QLQ-OG25 for esophageal cancer-specific QoL [22,23]. As part of the study protocol, patients will be asked to fill out QoL forms at the time of inclusion (baseline), at CRE-1 and at CRE-2.

## 4 Study endpoints

The primary endpoint of the TNT-OES-1 trial is the tolerability of the combined regimens, defined as the proportion of patients that complete the administration of the FLOT-CROSS regimen, *i.e.* receive all 4 cycles of FLOT and subsequently all 5 cycles of carboplatin/paclitaxel. Patients who complete both regimens but undergo limited dose reductions (*i.e.* up to 50% of the initial dose) or delays (*i.e.* not more than 3 weeks), will also be counted as patients completing the regimen.

Secondary endpoints are:

- The number of patients that have progressive disease after four cycles of FLOT chemotherapy and are thus unable to complete the FLOT-CROSS regimen;
- Serious adverse events and adverse events according to the CTCAE version 5.0 [19];
- The administered cumulatively dose of the used drugs in the FLOT regimen and the number of carboplatin/paclitaxel chemotherapy cycles administered as part of the CROSS regimen;
- The tolerability of the FLOT-CROSS regimen followed by a second dose of FLOT chemotherapy, defined as the number of patients who complete this regimen out of the patients starting the second round of FLOT chemotherapy;
- Disease control rate (DCR), defined as the percentage of patients with stable disease, partial response or complete response according to the RECIST criteria (version 1.1) at CREs [20];
- Objective response rate (ORR), defined as the percentage of patients with partial response or complete response according to the RECIST criteria (version 1.1) at CRE 1 and 2 [20];
- Progression-free survival (PFS), defined as time from inclusion to the date of the first documentation of progressive disease according to the RECIST criteria (version 1.1), date of death or date of last follow-up [20];
- Overall survival (OS), calculated from the date of inclusion to the date of death due to any cause or date of last follow-up;
- Clinically complete locoregional response (cCLR) rate, defined as the percentage of patients without residual locoregional disease at the second clinical response evaluation;
- Health-related quality of life (HRQoL), as measured by QoL questionnaires: EORTC QLQ-OG25 and EORTC-C30 [22,23];
- The proportion of patients who proceed to local therapy of metastases and/or esophagectomy;
- Surgical morbidity (including Clavien-Dindo score) and mortality (30-day, 90-day and/or in-hospital mortality), monitored within the Dutch upper gastrointestinal cancer audit (DUCA) surgical complications registry [24];

## 5 Patient safety

We incorporated stopping rules to be tested during the course of the trial. After the first 11 patients, we will evaluate the trial and at this time point we require that at least three patients have completed the combination of FLOT and CROSS successfully, i.e. have not experienced dose-limiting toxicity. If less than three patients were able to complete the FLOT-CROSS regimen, the trial will be stopped. If three or more patients were able to complete the regimen, another nine patients will be included up to the total of 20 patients.

## 6 Statistical analysis

### 6.1 Sample size calculation

This study combines two standard-of-care treatments into a sequential scheme. As reported 90% of patients starting preoperative FLOT chemotherapy for resectable gastric cancer is expected to complete all four preoperative cycles [8]. Also, 95% of patients undergoing only the CROSS regimen is expected to be able to complete that treatment [4]. The combination of FLOT and CROSS could lead to more toxicity. We therefore propose that further investigation of the combination of FLOT and CROSS treatment is only warranted if more than 70% of patients is able to complete the four cycles of FLOT plus the full CROSS regimen. The optimal Simon's two-stage design is used [25]. The null hypothesis that the true success rate (success defined as completion of all four FLOT cycles and all CRT sessions) is 0.40 will be tested against a one-sided alternative. The null hypothesis will be rejected if 12 or more successes are observed in 20 patients. This design yields a type I error rate of 0.05 and power of 80% when the true success rate is 0.70.

### 6.2 Data analysis

All analyses will be performed on the intention to treat population, with the exception of the safety endpoints, which will be performed in the per-protocol population (defined as the patients who receive at least 1 cycle of FLOT chemotherapy). Descriptive statistics will be used to analyze the primary endpoint tolerability. Progression-free survival and overall-survival will be analyzed by using the Kaplan-Meier method. Categorical variables will be presented as frequencies and percentages. QoL will be analyzed according to the EORCT manual [26]. Statistical analyses will be performed by using SPSS Statistics for Windows, version 25 (IBM Corp., Armink, N.Y., USA).

## 7 Discussion

In the present study, patients with adenocarcinoma of the esophagus or EGJ with oligometastases will be treated by the sequential FLOT-CROSS regimen. If this trial shows that the regimen is tolerable (*i.e.* more than 70% of patients are able to complete four cycles of FLOT and the complete CROSS regimen), a subsequent phase III trial is proposed to investigate the effectivity of this regimen in patients with oligometastatic disease and, as neoadjuvant treatment in patients with locally advanced, resectable non-metastatic esophageal or junctional adenocarcinoma. In these subsequent trials, the effectivity of the FLOT-CROSS regimen will be investigated.

The present study may also identify a treatment regimen with promising efficacy and worthy of further exploration in the metastatic setting. National and international guidelines consider systemic metastases as contraindication for treatment with curative intent [27,28]. Hence, patients with systemic disease are currently offered palliative therapy (systemic anti-tumor therapy, stent, or radiation) to relieve symptoms and/or prolong survival. The prognosis of these patients is poor with an estimated median survival of 6–10 months and a 2-year survival of less than 10% [29]. Recently, the phase-II FLOT-3-AIO trial showed a higher median overall survival in patients with limited metastatic disease who proceeded to surgery after 4 cycles of neoadjuvant FLOT compared to patients who did not undergo surgery [3]. Apart from the FLOT-3-AIO trial, most studies in patients with oligometastatic esophageal cancer have a retrospective study design, lack data on toxicity, feasibility and QoL, and well-defined selection criteria [30,31]. The combined FLOT-CROSS regimen as defined in the present study may open up different treatment options for patients with oligometastatic disease. For instance, those with a clinically complete response at the side of the primary tumor and regional lymph nodes can be offered local treatment of distant metastases (radiofrequency ablation, microwave ablation or metastasectomy), which may prolong life expectancy and improve QoL [[32], [33], [34]]. Also, in selected patients, treatment with curative intent may be offered. Lastly, QoL of patients may be improved as better local therapy may decrease symptomatic dysphagia and retrosternal pain. In addition, the prospective RENAISSANCE trial is currently investigating the effect of surgical intervention in previously untreated patients with limited metastatic disease [35]. Treatment consists of 4 cycles FLOT chemotherapy alone (or with trastuzumab if Her2+) and patients without disease progression are randomized to receive either additional chemotherapy or surgical resection of primary tumor and metastases, followed by subsequent chemotherapy. If this concept proves to be effective in terms of overall survival, this could lead to new therapeutic options for selected patients.

In this study, response to therapy will be assessed using the RECIST criteria (version 1.1) and evaluation of target lesions is described as: complete response (*i.e.* disappearance of all lesions), partial response (*i.e.* ≥ 30% decrease of the sum of longest diameters (SLD) of target lesions, no new lesions and no progression of non-target lesions), stable disease (*i.e.* no partial response but also no progression) or progressive disease (*i.e.* ≥ 20% increase of SLD, new lesions or progression of non-target lesions) [20]. It has been suggested that the RECIST criteria are not preferably used to evaluate response in the primary tumor. However, in metastatic disease, the RECIST criteria are most commonly used and makes it therefore possible to compare the results of our study with other studies.

The main risk for participants in this trial is death or morbidity from toxic side effects of the FLOT-CROSS combination. The toxicity of these regimens by themselves is well-known, and considered manageable in the non-metastatic (for the CROSS and FLOT regimens) and metastatic setting (for the FLOT regimen) [3,8,21]. However, the tolerability of the sequential FLOT-CROSS regimen in metastatic disease is still unknown. Multiple trials have investigated the combination of neoadjuvant chemotherapy followed by chemoradiotherapy. The TOPGEAR trial showed that preoperative chemotherapy (epirubicin, cisplatin and 5-FU) followed by chemoradiotherapy (5-FU with concurrent 45 Gy radiotherapy) is safe without a significant increase in toxicity or surgical morbidity compared to chemotherapy alone in patients with resectable gastric cancer [36]. The CRITICS-II trial is currently investigating three preoperative treatment arms in patients with resectable gastric adenocarcinoma in a phase-II, pick-the-winner design [37]. This study will add data to the question whether chemotherapy and chemoradiotherapy can be sequenced, but differs from the regimen used in our study as only two 3-weekly cycles of DOC are administered before chemoradiotherapy (compared to four 2-weekly cycles in our study). Other regimens investigating combined chemotherapy and chemoradiotherapy show a significantly higher rate of grade 3–4 leukopenia and dose-limiting toxicity (nausea or fever with neutropenia) in patients with locally advanced esophageal carcinoma [13,14]. These regimens were, however, substantially different from the FLOT-CROSS regimen which is investigated in the present trial.

Since preoperative FLOT could be completed by 90% of patients in the FLOT-4AIO trial, and since neoadjuvant CROSS could be completed by 95% of patients in the CROSS trial, we expect that the FLOT-CROSS regimen may be more tolerable than these two studies [8,21]. Tolerability may be improved if patients are properly selected prior to treatment, as proposed in the eligibility criteria of the present study.

In conclusion, if the present study shows that the FLOT-CROSS regimen is safe and tolerable, this regimen should be further investigated as a possible new standard of care for patients with locally advanced resectable non-metastatic adenocarcinoma. Moreover, this regimen may allow for better subsequent treatment options in patients with metastatic adenocarcinoma of the esophagus or esophagogastric junction.

## Funding

This research did not receive any specific grant from funding agencies in the public, commercial, or not-for-profit sectors.

## Disclosures

Bianca Mostert: Research funding Sanofi, Consultant Servier, Lilly

J. Jan B. van Lanschot: Research funding ZonMw and KWF

All remaining authors have declared no conflicts of interest.

## Declaration of competing interest

BM: Research funding 10.13039/100004339Sanofi, Consultant 10.13039/501100011725Servier, Lilly, J. Jan B. van Lanschot: Research funding 10.13039/501100001826ZonMw and KWF, All remaining authors have declared no conflicts of interest.
